# Approaches to the Origin of Life on Earth

**DOI:** 10.3390/life1010034

**Published:** 2011-11-18

**Authors:** Stuart A. Kauffman

**Affiliations:** 1 Tampere University of Technology, Tampere 33720, Finland; E-Mail: stukauffman@gmail.com; 2 University of Vermont, 85 South Prospect Street Burlington, VT 05405, USA

**Keywords:** origin of life, collectively autocatalytic sets, work cycles, maximum power efficiency

## Abstract

I discuss briefly the history of the origin of life field, focusing on the “Miller” era of prebiotic synthesis, through the “Orgel” era seeking enzyme free template replication of single stranded RNA or similar polynucleotides, to the RNA world era with one of its foci on a ribozyme with the capacity to act as a polymerase able to copy itself. I give the history of the independent invention in 1971 by T. Ganti, M. Eigen and myself of three alternative theories of the origin of molecular replication: the Chemotron, the Hypercycle, and Collectively Autocatalytic Sets, CAS, respectively. To date, only collectively autocatalytic DNA, RNA, and peptide sets have achieved molecular reproduction of polymers. Theoretical work and experimental work on CAS both support their plausibility as models of openly evolvable protocells, if housed in dividing compartments such as dividing liposomes. My own further hypothesis beyond that of CAS in themselves, of their formation as a phase transition in complex chemical reaction systems of substrates, reactions and products, where the molecules in the system are candidates to catalyze the very same reactions, now firmly established as theorems, awaits experimental proof using combinatorial chemistry to make libraries of stochastic DNA, RNA and/or polypeptides, or other classes of molecules to test the hypothesis that molecular polymer reproduction has emerged as a true phase transition in complex chemical reaction systems. I remark that my colleague Marc Ballivet of the University of Geneva and I, may have issued the first publications discussing what became combinatorial chemistry, in published issued patents in 1987, 1989 and later, in this field.

## 1. Early History

Before Louis Pasteur, there was no “problem of the origin of life”. It was widely thought that life arose spontaneously all the time, as witnessed by the sudden appearance of larvae in rotten wood after a rainfall. Pasteur won a prize with a brilliantly simple experiment. It was known that beakers with medium in them, left open to the air, soon had colonies of bacteria growing in them. Was this a spontaneous formation of life, as most thought? A prize was offered. Pasteur drew out a glass flask with an S shaped swan neck. He filled the beaker with medium, and the lower part of the neck with water, thereby blocking passage of air from the mouth of the neck to the medium. He waited. No bacteria grew in the flask. He, therefore, concluded that there was no spontaneous generation of life. “All life comes from life”, he declared.

With this beautiful result, the issue arose: how did life originate? Of course, in the Abrahamic tradition, God created life in Genesis. There the problem rested until the first half of the 20th Century when a Russian scientist, Oparin, studied jello-like coascervates, which were able to adsorb and desorb ions and small organic molecules from an aqueous environment. Life might, he hoped, start in such a way.

At about this time, J.B.S. Haldane proposed a model of the early oceans, or tidal pools or ponds, with a “primitive soup” of small organic molecules that might self organize into life; but how would such a soup form?

The famous next step was taken by Stanley Miller, in chemist Urey's laboratory at the University of California, Berkeley, when Miller was a graduate student. In a truly brave experiment, he created a beaker system to mimic early Earth's atmosphere, with ammonia, water and a few other simple molecules, an electric spark to simulate lightning, and an evaporation and recycling of the water in the beaker to mimic cloud formation and rain back into the beaker.

Miller left his mixture for several days. A brown scum formed at the bottom. On analysis it contained a number of the simple amino acids found in biological proteins. The conclusion was that the simple organic molecules of life might form under prebiotic conditions.

In the next decades enormous effort went into synthesis of virtually all the simple building organic molecules of life: sugars, lipids, nucleotides, amino acids. Typically, yields were low, and the reaction conditions forming each were different from those forming the others. This raised the question how the diversity of organic molecules synthesized in such a way might be assembled in one place for later biogenesis.

Meanwhile a second strand of work was underway. In the 1970s, a meteorite fell in Murchison, Australia. Called the Murchison meteorite, this material was a chondronaceoius meteorite rich with organic molecules, including amino acids and lipids. Later results found that the diversity of organic molecules in Murchison is over 14,000, with hundreds of thousands of others one reaction step away, which raises wonderful issues about the diversity of space chemistry, since the Murchison meteorite predates the formation of the earth.

Elsewhere, I have written about subcritical and supracritical chemical reaction networks. The latter, hypopopulated by mass, and evolving into an enormous “reaction graph” as driven by chemical reactions, starlight and other processes, is likely to be a vastly non-ergodic flow on this reaction graph, in which local fluctuations do not damp out as they do in simple reactions between, say two species, X and Y at millimolar concentrations, that flow to equilibrium with square root N fluctuations that do damp out. It was soon realized that bombardment of early Earth by meteorites might have brought prebiotic small organic molecules to earth by infall. At present, both scenarios, prebiotic synthesis on the early Earth and infall, are held to be co-contributors to the primitive soup of which Haldane spoke.

## 2. The Template Replicating Era

In announcing the structure of DNA, Watson and Crick, in fine British understatement, end the paper with, “It has not escaped our notice that the structure of the molecule suggests how it may replicate.” Nobody, looking at the exquisite symmetry of the DNA double helix, or its RNA double helix cousin, could fail to escape the thought that either single strand, let's call them Watson and Crick, specifies by base pairing, A:T, C:G for DNA, and A:U, C:G for RNA. Therefore, if an arbitrary single “Watson” strand template of RNA could line up free A, U, C and G nucleotides present in a medium, where A would hydrogen bond to a U in the RNA template, a C would hydrogen bond to a G in the template, and so on. Then the free bases would be lined up, ready to be bound by 3′ 5′ phosophdiester bonds into the new “Crick” strand. It was hoped that in the absence of any enzymes, but perhaps metals such as lead ions, or others, a second, new “Crick” strand would form the proper bonds, melt free from the old “Watson” strand, and the two single template strands would cycle again, forming a self reproducing, arbitrary nucleotide sequence, template pair of “Watson” and “Crick” template complements.

Leslie Orgel of the Salk Institute, and many others, tried to make this experiment work for about 40 years. It should have worked [1]; but at the time of this writing, no one has succeeded. Orgel explains some of the reasons for this failure: RNA nucleotides, when lined up by the Watson template, like to form 2′-5′ phosophodiester bonds thermodynamically. However, these polymers are unable to form an RNA double-stranded helix with the initial “Watson” strand. PolyC “Watson” strands can line up and synthesize about 14 G nucleotides. Unfortunately, the polyG polymer tends to fold and precipitate and so is unable to act as the second, new “Crick” strand. Failure with RNA led to work with similar polymers, such as PNA by Eschenmoser, but again without success.

## 3. Early Theories

In 1971, three authors independently proposed three different models for the origin of life. Timor Ganti at the Eotvos University of Budapest, proposed the “Chemotron” [2]. This was a bilipid layer hollow vesicle containing aqueous medium, like a liposome, containing a self replicating RNA double helix which was to replicate in the Orgel manner, and a simple metabolism bringing into the liposome the building blocks for the RNA polymer and the building blocks for the lipids forming the bilipid hollow vesicle. To his credit, Ganti was the first to bring together in one picture a minimal model of what would later be seen as satisfying at least minimal requirements for protolife.

In the same year, Nobel Laureate Manfred Eigen proposed the HyperCycle [3], based on Orgel-type replicating “Watson-Crick” RNA strands, but with a set of such N pairs, 1,2,3,..N. The additional idea was that replicating pair 1 would catalyze or help the replication of 2, 2 would help 3 and so on, until N closed the “hypercycle” and helped replicate 1. The Hypercycle was the subject of intense mathematical investigation for its proliferative capacities, its stability and instability to alterations in the loop, 1,2,…N, shortening the loop and thus out-replicating the longer hypercycle, and other fine work.

The third model, that of “Collectively Autocatalytic Sets”, CAS, was proposed the same year by myself [4,5,6]. My own initial motivation was the question as to whether if the constants of nature in physics were altered, would life still arise. In modern terms, if we were in a universe such as the hoped for multiverse of physicists, where chemistry was different, and the DNA double helix and RNA double helix could not form, but some altered chemistry existed, would life be ruled out?

I did not want this to be true, so I sought a route to molecular self reproduction, fully independent of the Orgel and “Watson-Crick” template replication. It was obvious that life can speed up chemical reactions, *i.e.* catalysis, or “kinetic control”, compared to background reactions in some complex reaction mixture. What was needed? A set of organic molecules forming a reaction network; a set of input “food” organic molecules to drive the system away from chemical equilibrium; and, critically, a set of organic molecules within the reaction network such that each served as a catalyst to one or more reactions and such that the set of molecules collectively catalyzed all the last steps in the formation of each member of what I came to call “collectively autocatalytic sets,” CAS. Thus I introduced the concept of molecular reproduction via a collectively autocatalytic set, rather than by template replication of an RNA like molecule.

A simple case of such a system would be one molecule, A, which catalyzed its own formation from some precursors to A.

However, the first essential new idea was that two molecules, A and B, might have the property that A catalyzed the formation of B from B precursors, while B catalyzed the formation of A from A precursors. Note that this is the simplest COLLECTIVELY AUTOCATALYTIC SET, and NO molecule in the set catalyzes its own formation. Rather the set as a WHOLE catalyzes its formation from precursors.

In addition, beyond a single autocatalytic molecule, A above, this is the simplest case of a kind of “catalytic functional closure”. All the reactions that need to be catalyzed are catalyzed such that the set reproduces itself. This is a simplest model of a more general functional closure in dividing bacteria or other living cells, where an unspecified set of functionalities are all accomplished and the cell is, in fact also a collectively autocatalytic set. No molecule in your cell catalyzes its own formation.

The next basic issue was this: Under what conditions would one expect such a collectively autocatalytic set to form? Obviously this question depends upon the molecular species present, the reactions that can take place among them, the abundance of food molecules, and, most critically, the distribution of which molecules in the system catalyzed which reactions. Were we to know this for any set of molecules, we could determine whether it contained one or more collectively autocatalytic sets.

In 1971, and now, we do not know which molecules catalyze which reactions. In my first model, I made the radically simple assumptions that the molecules were polymers of two types of monomers, A and B, say two amino acids, or later, two nucelotides. These could undergo only cleavage and ligation reactions. Monomers, A and B, and dimers, AA, AB, BA, and BB might serve as sustained food inputs to the system. Given this, the reaction network among the molecules could be determined. I called this network a “reaction graph,” consisting of two types of nodes, the first representing molecules, the second representing, as boxes, reactions. Arrows led from substrate molecules to the respective reaction box, and further arrows from there to the product(s). The directions of the arrows serve only to identify substrates and products, not to direct a chemical reaction which depends upon the direction of displacement of the reaction from its equilibrium.

The hardest part was to model which molecule catalyzed which reaction. I initially used the simplest assumption. Each molecule has a fixed probability, Pcat, of catalyzing any given reaction. I then assumed for simplicity that uncatalyzed reactions occurred so slowly that they did not matter, and that catalyzed reactions were “fast.”

Given a value for the probability of catalysis by a polymer, Pcat, it was then simple to simulate, and later prove, theorems that showed the following: (1) As the diversity of molecules in the system increases, the diversity of reactions among them increases EVEN FASTER. So the ratio of “reactions” to “polymers” increases; (2) Given a fixed probability of catalysis, as the diversity of polymers increases, more and more reactions are catalyzed. At first single isolated reactions are catalyzed, then pairs of adjoining reactions are catalyzed, then clusters of neighboring reactions are catalyzed. Then, in a phase transition that is the analogue to the famous emergence of a Giant Component in a random Erdos Renyi Graph [4], when a few more reactions are catalyzed as diversity increases, a Giant connected component of a network of catalyzed reactions forms. It can be shown that with probability 1.0, the system will contain a collectively autocatalytic set.

I want to stress a number of features of this model, now well analyzed, with theorems establishing the claims above, and many computer simulations confirming it.

(1) The model does not depend upon the specifics of which molecules catalyze which reactions. The formation of a collectively autocatalytic set is an emergent property of the combinatorial character of molecules, such that the ratio of reactions to molecules generically increases as the diversity of molecules increases. Indeed, were two substrates—two product reactions—allowed, the formation of CAS is even easier;
(2) In an important sense, this theory is independent of the underlying physics and not reducible to any specific physics, e.g., that of our universe. Reductionism in science has nothing to do with the adequacy of this theory, which might hold in “neighboring universes” in which chemistry and catalysis could still occur. Indeed, given an experimental CAS, physicists could reductively explain the reproduction of that specific CAS; but such an explanation would not be the overarching theory of the emergence of collectively autocatalytic sets given entities called molecules, reactions and catalysis;
(3) From this view, the emergence of self reproduction is to be expected in sufficiently diverse chemical systems. Life, in the sense of molecular reproduction, may be expected and widespread in a universe with many solar systems.

I return later to subsequent developments of this model, suffice to say here that a later version was published in 1986, [5], and is reported in my book Origins of Order, 1993, [6]. In that book I reported early work with Richard Bagley based on a somewhat more realistic model of the requirement for catalysis: In this simple model, a condition that any polymer was a candidate to be a catalyst remained Pcat, but in addition, if it could be a catalyst, its left and right “ends” had to “string match” the right and left terminal monomers of those of its two substrate(s) for a ligation reaction, with allowed mismatches so that a catalyst might catalyze similar reactions. Thus, this model goes beyond “only” the Pcat of the first model, and allows a subset of models to have a chance to be catalysts, and, by allowing mismatches, allows a given catalyst to catalyze a FAMILY of similar reactions. This is a much more realistic model and yielded much the same results of a phase transition at a critical diversity of polymers and candidate catalysts to collectively autocatalytic sets. Both models ignore inhibition of catalysis, compartmentalization, and evolution, subjects which are returned to below.

## 4. The RNA World Era

In the late 1980s, Cech [7] and Altman discovered that RNA introns, sequences within RNA transcripts of eukaryotes that are spliced out of that RNA to make mature mRNA, could act as ribozymes and catalyze the splicing reaction itself. The two won a Nobel prize for the discovery. In turn, the discovery that the same class of molecules, RNA, could carry genetic information, as in RNA viruses and messenger RNA, mRNA, and also catalyze reactions triggered a huge response in the biological community. In cells, most catalysts are proteins, but proteins do not carry genetic, arbitrary template replicating information.

Signs of very early RNA sequences in cells were found. Most notably, the ribozyme, responsible for protein synthesis by reading mRNA triplet codons and using “transfer RNAs” each attached to the amino acid coded for by that codon, which has RNA sequences as its ribozyme catalytic site to carry out protein synthesis. Also, an evolved RNA has been found able to bind two amino acids into a dipeptide.

This has given rise to the RNA World Era, in which most workers in the origin of life field have come to believe that at some stage, life was based only or predominately on RNA. Here there are two alternative views. In the first, minority view, RNA ribozymes might form collectively autocatalytic sets. In the second, the existence of a ribozyme able to copy itself and any other arbitrary RNA sequence, in a sequence specific manner, hence a ribozyme “polymerase”, might exist and have been the first “living molecule.” David Bartel at the Whitehead has evolved, from a set of 10 to the 15th random RNA sequences, one that is able to template replicate 14 nucleotides of a given sequences, showing that a ribozyme polymerase may indeed be possible.

However, I have always worried about the evolutionary stability of such a hard to find ribozyme polymerase. Consider it reproduces itself with errors. The new copies would tend to reproduce themselves with even more errors leading, perhaps to a runaway “error catastrophe”, first thought of by Orgel in a different context. No work has been done to study the selective conditions that might stabilize such a ribozyme molecule against such an error catastrophe.

## 5. The Lipid First World

Luigi Luisi [8] and David Deamer [9] are pioneers in research on how life might have started with liposomes that form from lipids, which have water-loving and water-hating ends, hydrophilic and hydrophobic ends. Placed in water or buffer, the lipids form a bilipid membrane in which the hydrophobic ends of two lipids point “up” and “down” toward the aqueous phase, while the hydrophobic, oily ends nestle next to one another. Once this membrane forms, it tends to form further into hollow vesicles containing the aqueous medium.

Liposomes are candidates for the “containers” described by Ganti [10]. Some means of isolating any polymer reproducing system, so its constituents do not diffuse out of reaction contact with one another, is probably needed and yields protocells. Liposomes, very similar to cell membranes, are an obvious candidate and manifest interesting properties. Luisi showed that in appropriate circumstances liposomes could grow in size then divide into two smaller liposomes in repeating cycles [8]. Thus liposomes are simple self-reproducing molecular structures that separate an “inside” from an “outside” environment.

David Deamer has shown that lipids from the Murchison meterorite can form liposomes, and that liposomes subjected to wet dry cycles can take small proteins called peptides, DNA and RNA and other polymers into their interior. A consensus is emerging that budding liposomes are a critical component in the emergence of protolife.

## 6. Metabolism First Theories

All extant earth life is based on a common core of metabolism centered around the tricarboxylic, TCA, cycle. Harold Morowitz and others [11] have realized that if this cycle of reactions is run in the reverse direction, two copies of one of the members of the cycle are created for each “turn” of the TCA cycle, making the reaction cycle as a whole “autocatalytic”, in the sense of producing two copies of one of its members. Thus the cycle of reactions as a whole acts as a catalyst for that molecular species.

These facts are part of the theory that life starts with a connected metabolism, perhaps adding the formation of a polymer enzyme that can speed up other reactions in a reaction system.

It is conceivable that if a cycle of reactions can be a catalyst for a given reaction forming a specific molecule, there might be an autocatalytic cycle of cycles such that all reactions are catalyzed by cycles of reactions. No one knows. Or it may be that speeding up a few reactions, given sustained inputs of food stuff molecules, is enough for reproduction of the system of reacting molecules, particularly if placed inside a dividing liposome.

## 7. Experimental Work on Collectively Autocatalytic Sets

In the early 1990s, Guenter von Kiedrowski [12] created the first reproducing autocatalytic molecule, a single stranded DNA molecule with 6 nucleotides, for simplicity CCCGGG. von Kiedrwoski used two trimers, GGG and CCC, and hoped the hexamer, CCCGGG would Watson-Crick base pair bind the two trimers, GGG to the left CCC end of the hexamer, and CCC to the GGG end of the hexamer, then form a proper 3′5′ phosophdiester bond. Because of the left right, or 3′5′ assymetry of the hexamer and two trimers, the newly formed hexamer is identical 3′5′ to the original hexamer, 3′CCCGGG5′. The experiment worked, producing the world's first autocatalytic molecule—a triumph. von Kiedrowski's experiment produced, not an arbitrary DNA sequence template, replicating itself by Watson-Crick base pairing, as Orgel envisaged. Rather, the hexamer, CCCGGG acts as a simple enzyme “ligase” to bind two specific trimers, GGG and CCC, and ligate them to form a second sequence GGGCCC. Nor is this the template replication of an arbitrary DNA or RNA sequence that Bartel sought. Rather, it is an autocatalytic reaction through which the hexamer forms a second copy of itself from fragments of itself.

Soon von Kiedrowski had created the world's first collectively autocatalytic set (CAS), in which two hexamers, A and B, had the property that A catalyzed by ligation the formation of B from B fragments, and B catalyzed the formation of A from A fragments. When I first met Guenter, we shared a bottle of champagne to celebrate his successful experiment.

## 8. Peptide Collectively Autocatalytic Sets

In my 1971 paper, I focused on protein or peptide collectively autocatalytic sets, in part because proteins were known enzymes and catalyzed reactions. My work was essentially ignored, in part because the biological world was so fascinated with template Watson-Crick like replication. There is no obvious way a protein, a sequence of 20 kinds of amino acids that folds into a structure, might specify its specific sequence and reproduce in a template like sequential synthesis fashion. This “template replication” concept is fixed on the idea of reproducing the sequence of a specific arbitrary protein by catalyzing a sequence of amino acid addition reactions in a growing polypeptide chain, by which a copy of the initial arbitrary amino acid sequence polypeptide forms.

In 1995, Reza Ghadiri made the first autocatalytic peptide. His work mirrored von Kiedrowski and my hopes for peptide ACS. Ghadiri used a 32 amino acid sequence from a zinc finger protein, forming an alpha helix that coils back on itself to form a coiled coil. He reasoned that two fragments of this sequence, each long enough to form an alpha helix, might be recognized and bound by the 32 amino acid sequence, then ligated to form a proper peptide bond between the two fragments. Ghadiri used 15 mer and 17 fragments that together constituted the entire 32 amino acid sequence, activated these fragments chemically to drive the reactions in the direction of ligation, added the 32 long peptide, and it worked. The peptide did ligate the 15 and 17 fragments of itself into a second copy of the same peptide. Ghadiri showed, once and for all, that molecular self reproduction need not be based on template replication like that of DNA and RNA.

Soon Ghadiri had created the world's first collectively autocatalytic peptide set, where A ligated fragments of B to form B, and B ligated fragments of A to form A. At present, Ghadiri and his former postdoctoral fellow Gonen Ashkanazi [13], have a 9 peptide collectively autocatalytic system. In addition, Ashkanazi has engineered these so they can realize all 16 logical gate or Boolean functions of two molecular inputs. The way is open to study not only autocatalytic sets, but the dynamics of such catalytic networks, such as multiple dynamical attractors, and the relation between the possibly complex dynamics and the efficiency of reproduction.

## 9. RNA Collectively Autocatalytic Sets

Recently, Lam and Joyce at Scripps have succeeded in finding two pairs of ribozyme collectively autocatalytic sets, *i.e.*, A catalyzes B and B catalyses A, C catalyses D and D catalyzes C [14].

## 10. Experimental Work towards Peptide Collectively Autocatalytic Sets

Given my 1971 model, the primary question was: what is the probability that an arbitrary protein or peptide might catalyze an arbitrary reaction, *i.e.*, what is Pcat? A decade later, I heard in a lecture that upon deletion of a bacterial enzyme, beta galactosidase, from the bacterium E coli, the bacteria, if grown on the substrate for beta galactosidase, namely the sugar lactose, could evolve a new enzyme able to catalyse metabolism of lactose. I was inspired to realize that I could test my question about Pcat by making millions of stochastic DNA sequences, cloning them into bacteria, and selecting for the capacity to catalyze arbitrary reactions. I realized also that if I wanted to mimic the shape of estrogen I could take the estrogen receptor, think of estrogen as a key, and the receptor as a lock, screen millions of random peptides for those bound by the receptor, and obtain candidate mimics of estrogens. Hence I could seek drugs.

With Marc Ballivet at the University of Geneva in 1985, we carried out the first synthesis of a stochastic DNA “library” of tens of thousands of random DNA sequences, cloned into bacterial vectors or phage vectors, showed that we had made such libraries and that they encoded “fusion proteins” due to the insertion of the random DNA sequences into a gene coding for beta galactosidase. Ballivet and I filed the first patent in 1985 on what later became combinatorial chemistry, [15,16].

Ballivet had envisioned cloning random DNA into a gene expressed on the outside of a virus and screening viruses, *i.e.*, phage display, work neither he nor I performed. In 1990, this area broke open when George Smith at the University of Missouri independently thought of Ballivet's idea, cloned random DNA sequences into a bacterial virus, “displayed” on its surface, and showed that if 20 million were screened for binding to an arbitrary ligand, a “monoclonal antibody” 19 quite different random peptides were found [17]. From this has grown our current knowledge that ligand binding of a random peptide to an arbitrary ligand is about one in a million.

Since binding is a step toward catalysis, one in a million is a lower estimate, bound on the chance that a random peptide catalyzes a random reaction. However, that chance, in turn, depends upon the chance that a random polypeptide folds reliably into a three dimensional shape. In 1994, Thomas LaBean in my lab at the University of Pennsylvania, completed Ph.D. work showing that some fraction of a random peptide library did fold into reasonably compact 3D structures [18]. Subsequently, Luisi has recently shown that such “never before born” peptides have about 30% chance to fold well. These would now be reasonable candidates for catalysts. Yomo *et al.* have shown that long random peptides can evolve to catalyze reactions [19].

It has always seemed likely to me that peptides are more chemically diverse than RNA sequences, hence might form CAS more readily. In partial support of this, Jack Szostak and Andrew Ellington in 1990 showed that a library of 10 to the 14 random single stranded sequences could be screened and about one in a hundred million would bind to an arbitrary ligand [20]. These sequences are now known as “aptomers.” So, roughly, binding a ligand is 100 fold easier for random peptides than for random RNA sequences.

## 11. What is the Status of the Theory of CAS as Emergent in Sufficiently Diverse Chemical Libraries?

The essential experimental and theoretical avenues here include: (1) experimental assessment of the distribution of the probability of catalysis by peptides, or RNA, or other molecular species, as a function of the length or number of atoms per molecular species; (2) Careful assessment for a complex reaction mixture of the probability that it contains one or more CAS, each with one or more dynamical attractors due to inhibition of catalysis as well as catalysis; (3) Thus the ease of detecting one exponentially reproducing autocatalytic set in a chemostat experiment, or the coexistence of several CAS given subexponential growth of each; (4) Possible use of the concept of dynamic combinatorial libraries to generate a flow in a reaction network toward a CAS; (5) We can, in this way, tune the diversity of, say, stochastic peptide sequences of different lengths. In assessing the probability of catalysis of, say, random peptides we can use a stable analogue of a transition state of a reaction and select for peptides, as a function of their length, that bind the transition state and catalyze the reaction in question; (6) Alternatively we can present a population of stochastic peptides with a population of substrates, say peptides, representing a diversity of reactions, and ask when at least one or a few reactions are catalyzed. This would allow us to estimate the probability of catalysis of a reaction by a random peptide. Concretely, consider using a library of peptides length 10, 20, 30,…., and tuning the diversity of each from 1, 10, 100, 1,000, 10,000, plotted on the X axis as candidate catalysts. On the Y axis use the same peptides as substrates, 1, 10, 100, 1,000…. Now, if we consider only ligation reactions, the number of distinct ligation reactions is the square of the diversity of substrate peptides. Consider confronting two random peptides as substrates with one random peptide as candidate catalysis. If the probability of catalysis is one in a billion, almost certainly no reaction will occur. Conversely let 10,000 substrate peptides, representing 10 to the 8th power reactions be mixed with 10 to the 7th peptides, each having a probability of one in a billion to catalyze a reaction. Then we expect 10 to the 8 × 10 to the 7 divided by 10 to the 9th = 10 to the 6th reactions to be catalyzed forming 10 to the 6th ligation products. However, these 10 to the 6 new products now correspond to about 10 to the 12th reactions, so the next expected number of catalyzed reactions is 10 to the 12 × 10 to the 7 divided by 10 to the 9th = 10 to the 10 products! The products formed should, in short, explode in diversity. Thus a roughly hyperbolic curve in this X Y space separates a region below the curve which is subcritical, from that above which is supracritical, and should exhibit the catalysis of an increasing diversity of products. A supracritical system may well contain one or more collectively autocatalytic sets; (7) Theoretical work, below, shows such sets can evolve, if they evolve toward higher specificity and higher catalytic efficiency CAS, they may cut off the supracritical explosion and catalyze only their own formation. Chemostat experiments might detect such CAS.

## 12. Further Theoretical Work on Collectively Autocatalytic Sets

I had studied neither inhibition of catalysis, nor compartmentalization of CAS, nor the capacity to evolve, [5,6] nor, what, mathematically constitutes minimal requirements for the emergence of CAS in a peptide library. Recently W. Hordijk *et al.* have shown that achieving CAS is very much easier than originally thought [21].

With respect to the capacity of CAS to evolve, Doyne Farmer, Rick Bagley and Walter Fontana showed in the late 1980s that CAS could indeed evolve [22,24]. More recently, Eors Szathmary and colleagues have completed a careful body of work that combines compartmentalization of CAS in budding liposomes, competitive and non-competitive inhibition of catalysis, and evolution of CAS in dividing compartments. Their conclusion is that under these conditions, compartmentalized CAS can undergo open-ended evolution and are plausible models for protocells [23]. Further work by Roberto Serra has shown that collectively autocatalytic sets in dividing compartments synchronize compartmental division and CAS reproduction [24], which fits well with the results of Szathmary *et al.* Furthermore, Serra is using chemical master equations with the Gillespie algorithm, allowing fine-grained analysis of the dynamics of CAS as a function of catalysis and competitive and non-competitive inhibition of catalysis. For example, he shows that multiple dynamical attractors are found, and under which conditions they are found. This work is poised to join that of Ashkanazi and his engineered “logic gates” for 9 peptide CAS. In addition, Serra has early interesting results on energy utilization in CAS that may ultimately be tied to the issues of the next section. Kaneko *et al.* have studied molecular species abundances in CAS and found a power law distribution, [24].

## 13. Linking Exergonic and Endergonic Processes Forming Work Cycles

An essential feature of the CAS above is that they can be purely exergonic. Real cells link exergonic and endergonic reactions in complex webs of reactions, and perform work cycles such as chemo-osmotic pumps.

- Consider a hypothetical world in which only exergonic reactions can be coupled;
- Consider a hypothetical world in which exergonic and endergonic reactions can be coupled.

Almost a Theorem: The richness of the web of coupled reactions is far greater if exergonic and endergonic reactions can be coupled, than if only exergonic reactions can couple. In turn, the more complex the web of coupled reactions, together with the chance that molecules in the web are catalysts for the same reactions, the easier it is to form collectively autocatalytic sets. There may well be a selective advantage in the formation of CAS to link exergonic and endergonic reactions.

The more subtle point made in my book, Investigations, [26,28], is portrayed by a whimsical machine linking exergonic and endergonic processes: A cannon is poised to fire a cannon ball. A charge is at the base of the cannon. A cannon ball is loaded just beyond the charge. With foresight, a well has been dug some 100 yards away and filled with water. Straddling the well is a paddle wheel. Tied to its rim is a rope leading down to a pail handle attached to a water-filled pail in the water bottom of the well. The charge is exploded in an exergonic process. The cannon ball flies in an endergonic process and hits a paddle on the paddle wheel, which spins in an endergonic process, winding up the rope in an endergonic process, lifting the pail of water in an endergonic process. The pail tips over the rim of the wheel and water from it falls by gravity in an exergonic process into a funnel and tube leading down hill to my very own bean field. In a further exergonic process, the water flows down the tube, endergonically opens a flap valve, exergonically flows to my bean field and waters my bean field. I am very proud of my machine.

At the end of this venture, the cannon ball lies in a field a dozen yards from the paddle wheel and the pail lies on the ground beside the well. Now consider “feeding” this system by adding a second explosive charge to the now empty cannon. Can we water the bean field again? NO.

In order to water my bean field again, I have to do work to fetch the cannon ball and put it into the cannon just beyond the new second explosive charge, and replace the pail into the well where it fills again with water. Now I can water the bean field again.

Note though, that in my doing work to replace the cannon ball and put the pail in the well, I have participated, enabling the total system to complete a work cycle. My role could have been played by gears and chains and escapements as in a real engine.

Thus a central point. IF exergonic and endergonic processes are linked, there is no point in eating unless a work cycle is completed. It is useless to take in food, or a renewed energy source, (the second explosive charge placed in the base of the cannon), if a work cycle is not completed. Work cycles are necessary to the efficacy of feeding if exergonic and endergonic processes are linked.

## 14. Energy, Work Cycles, Power Efficiency, and an Optimal Displacement from Thermodynamic Equilibrium

The above scenario of exergonic CAS is, I propose, insufficient. Real cells need both energy, and to perform thermodynamic work cycles by which spontaneous (exergonic) and non-spontaneous (endergonic) processes are linked into large webs of cyclic and cross cyclic processes, which are in fact work cycles.

At present we know that simple substances, such as pyrophosphate, might serve as driving energy sources; but that might still be a trivial extension from purely exergonic CAS, driven by pyrophosphate. Real cells use proton gradients, e.g., mitochondria use ATP to drive endergonic reactions. We have little theory about the emergence of work cycles in protolife, although we know that life is a non-equilibrium process. From the above, if we link exergonic and endergonic processes, there must be completed work cycles for food to be useful. As Schrodinger said in *What is Life?* we eat negentropy and excrete entropy. However, Schrodinger missed the need for work cycles if exergonic and endergonic processes are linked. We have had no theory for how far-from-equilibrium cells do work cycles. Carnot showed maximum energy efficiency for work cycles if performed infinitely slowly, *i.e.*, adiabatically. Although, if the work cycle is needed for cell or protocell reproduction, such a cell will lose the Darwinian race, implying that energy efficiency must be the wrong concept.

A start towards such a theory is now underway. Consider an automobile. At what velocity is maximum fuel efficiency found? That is, at what speed is miles per gallon, or kilometers per liter, maximized? At 2 miles per hour, 47 miles per hour, or 2,005 miles per hour? We all know it is at about 47 miles per hour. At a constant velocity a car experiences the friction of the road. Air friction must be overcome, so work must be done to sustain a constant velocity. Work per unit time, equals power. A constant power is required to sustain a fixed velocity. Then at 47 miles per hour a car optimizes its power efficiency per unit fuel.

Now consider cells. It takes work to gather food which provides energy for work. One would think that after 3.7 billion years of life, cells would be good at maximizing the work they can perform building themselves, at the price of doing work to gather the energy to do that work.

The analogue of miles per hour for an automobile is biomass production per unit food, e.g., glucose, used which is again a power efficiency per unit fuel.

With Tommi Aho and Olli Yli-Harja at the Tampere University of Technology Finland, we [26] have taken a model of *E. coli* metabolism, calculated biomass production rate per unit fuel use rate, plotted on the Y axis *versus* fuel (glucose) use rate on the X axis. We find a unimodal distribution with a maximum at a finite rate of glucose utilization per unit time, a power efficiency maximization point, which seems interesting because it picks out a preferred displacement from chemical equilibrium, maximizing the efficiency of cell reproduction per unit fuel used.

This criterion is related to K, not R selection in ecology, *i.e.*, selection, not for a high reproduction rate, but for sustained reproduction when food resources are limited. If this is a general condition in life, for example in bacterial colonies, or ecosystems, we may have found a principle for an optimal power efficiency in the work cycles of life.

Current work shows that this theory fits the empirical data available somewhat better than the standard model of “most rapid growth.” More bacteria tune their growth rate based on their density by quorum sensing, perhaps bringing bacterial colonies to a power efficiency optimum.

When power efficiency is maximized, heat production should be minimized such that, a maximum amount of the energy available to cells is going into reproduction of cells, not into waste heat. If this is correct, life does NOT maximize entropy production and flattening energy gradients, but maximizes power efficiency per unit food utilized, under K selection, at a specifiable optimal displacement from equilibrium where it may minimize entropy production compared to reproduction rate.

## 15. Chirality

The early formation of collectively autocatalytic sets, say of peptides and RNA, may be necessary for the evolution of chirality in these polymers. All life shares homochiral, L, proteins, and D RNA and DNA. Why? While brilliant work shows that chiral symmetry breaking reactions can produce enantiomeric excess of one monomer, so that symmetry breaking can go either to L or R. Among M such monomers, how is a set of M identical L monomers to be formed if symmetry breaking is an independent event among the M? Further, work shows that small peptides can form as chiral peptides, but the symmetry can be broken L or D, for any such peptide. Among a set of M distinct chiral peptides, each homochiral, why should all M be of the same chirality, L or D? Conversely the simple hypothesis that homochrial polymers, DNA, RNA, or polypeptides are either more stable or function better than racemic polymers, suggests that collectively autocatalytic sets of such polymers in which all the polymers of a given type, e.g., polypeptides, are of the same chirality, say L, would be selectively advantageous to the CAS as a whole. Indeed, were some of the polymers D and some L, ligation and cleavage reactions among them would make less functional racemic peptides. This may suggest a role for homochiral CAS in the onset of chirality, but needs to explain how such a system faced with small homochiral and racemic food, avoids the latter, and how a single homochorial CAS can evolve into a world of CAS all of the same chirality.

## 16. Conclusion

I have reviewed, in a fairly broad way, the history and current status of theories and experimental work on Collective Autocatalytic Sets (CAS) in the context of emergent mechanisms for the origin of life. I believe the field is poised to explode in the near future, perhaps to create self-reproducing, evolving, *de novo* life forms in the next few decades. From these examples of evolving protolife, the later evolution of life based on DNA, RNA and encoded protein synthesis remarkably frees collectively autocatalytic modern cells to explore protein and RNA sequence space for novel functions at many levels. This further evolution is an additional hard task.

We now have the tools to understand much about the origin of life as an emergent property of complex chemical reaction networks and the three dimensional spatial structures they can form.
